# Can we learn from an imagined ransomware attack on a hospital at home platform?

**DOI:** 10.1038/s41746-024-01044-5

**Published:** 2024-03-20

**Authors:** Stephen Gilbert, Francesco Ricciardi, Tauseef Mehrali, Constantinos Patsakis

**Affiliations:** 1 Else Kröner Fresenius Center for Digital Health, TUD Dresden University of Technology, Dresden, Germany; 2 IRCCS Casa Sollievo della Sofferenza, San Giovanni Rotondo, Italy; 3 Ada Health GmbH, Berlin, Germany; 4 University of Piraeus and Athena Research Center, Dimitriou, Greece

**Keywords:** Health policy, Health services

## Abstract

The hospital at home concept integrates key digital medicine technologies and concepts in a single platform approach, with telemedicine, wearables, and sensors. It could bring benefits to patients, who face lower risks from hospital infections and who want to be at home with their loved ones. Moreover, it may lead to efficiency savings, through its seamless integration of data flows, and therefore is likely to be an increasingly implemented model. But what happens when the platform succumbs to exploited platform/infrastructure vulnerabilities or cyber attacks like ransomware that have been weaponized to bring networked systems crashing down? Exploring the attack modes and their consequences could help prioritize adequate safeguards.

How critical is cybersecurity for digital medicine patient sensors, such as those used in remote patient monitoring? During the COVID-19 pandemic and subsequently, the hospital at home (HaH) concept has been increasingly adopted, particularly in the US, Switzerland, and the UK^1^. It is an innovative model that provides hospital-level care in the patient’s home, supported by digital systems, remote monitoring, and telemedicine^2^. It can be used as an alternative to admission or to enable earlier discharge^3^, and it has been proposed as a substitute for acute care in hospitals for specific patient populations, who would traditionally have required close hospital observation. This model also allows for a more rational use of health professionals and better addresses the shortage of professional health workers. The following simulated scenario, which has been designed with reference to realistic HaH implementations^4–7^ and reports of attacks on hospital systems^8,9^, explores a possible ransomware attack and its consequences for patient safety and privacy in a fictional implementation of the HaH concept. The scenario is described as a sequence of events in different settings relevant to the HaH, the interconnection of which becomes clear as the narrative progresses.

## ‘Ward@Home’ launch and development

The following events are reported in chronological order. This simulated scenario is entirely hypothetical and fictitious. Any similarity to actual persons, entities, or organizations is purely coincidental.

### System launch, March 15, 2023, metropolitan area, somewhere in continental Europe

Patients in *Ward@Home*: 0

**Fig. 1:**
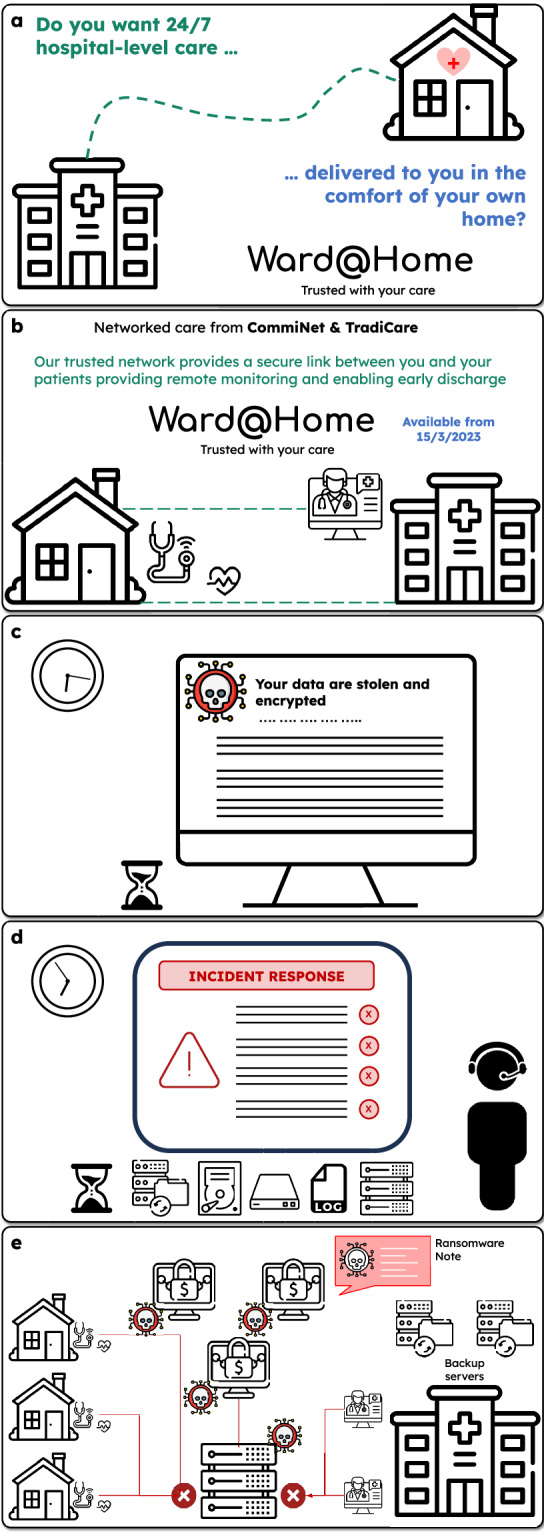
Storyboard of events. **a** Launch of Ward@Home service and description for patients. **b** Launch of Ward@Home service and description for physicians. **c** Ransomware attack notification message. **d** An Incident Room (IR) is established according to the Ward@Home cybersecurity procedures. **e** Description of the Ransomware attack and its effects on the Ward@Home network.

National Press office, CommiNet: “*CommiNet and TradiCare launch the* Ward@Home *program to transform regional health services*. Our partnership will deliver Ward@Home solutions via our unique virtual care platform (Fig. 1a). We use AI to collect and monitor health information remotely, working with our community care professionals. The platform detects problems with a patient in real-time, predicts the risk of worsening conditions, and notifies the patient, carers, and health care professionals, as required. Today’s launch is a proud moment for CommiNet and TradiCare, and our ‘doctor-led, digitally enhanced’ strategy.”

### Pre-launch review, March 16, 2023, CommiNet corporate HQ

Patients in Ward@Home: 0

*Meeting: ‘*Ward@Home’ *platform* pre-launch system review.

*Present: Senior executive and contributing project/clinical teams CommiNet & TradiCare*,

*Presentation, ‘Summary of Risk Management’, CommiNet VP of Regulatory, Quality and Compliance (VP RQC): “In the risk management report, you can see that all risks have been mitigated. All residual risks are assessed as acceptable, and the benefit-risk assessment shows a positive ratio of risk versus benefit … Indeed, we are confident that the patients in Ward@Home will receive equivalent, if not better, monitoring than in standard hospital care, particularly at night and weekends. The system is fully compliant with GDPR*.”

Question: IT Systems Responsible Person, TradiCare: “How has the risk of extended system downtime been mitigated?”

Answer CommiNet *VP RQC*: “*Our TightNet™ network system architecture has achieved 99.999% uptime across all our regional networks for 5 years, and the ‘Ward@Home’ platform software is verified and validated, passing all test sets and is compliant with the state-of-the-art cybersecurity standards*.

Answer: Chief Transformation Officer, TradiCare: “We can switch back to the current standard of care if we experience any unexpected teething problem. We will be closely monitoring the platform as it goes live. Our community teams are well-trained, and the 24/7 operation center is adequately staffed to sort out any launch issues. Our backup policies keep incremental backups daily, and we have provisioned long-term offline backups. This allows us to revert our systems almost instantly to any secure state in the past, should it be necessary.”

### TradiCare Annual General Meeting, January 24, 2024

Patients in Ward@Home: 573

Presentation, ‘Corporate Statement’, CEO TradiCare: “The healthcare sector has been under significant pressure in 2023. As a sector, we face a convergence of cost pressures and tightness in the clinical labor market with a shortage of both doctors and paramedics. TradiCare has a better economic outlook than similar care systems due to our pioneering technology adoption. We will continue to leverage our digital transformation program to deliver significant cost savings and allocate our clinical personnel in the best way possible.

## ‘Ward@Home’ ransomware attack, July 20, 2025, Sunday

Patients in Ward@Home: 7597, of which 2991 have the status ‘close observation’

Staff at operation center: 13

Community care staff: 23 (attending patients); 13 (available)

### Time: 00:06:17. Ambient temperature: 23 °C

**Fig. 2:**
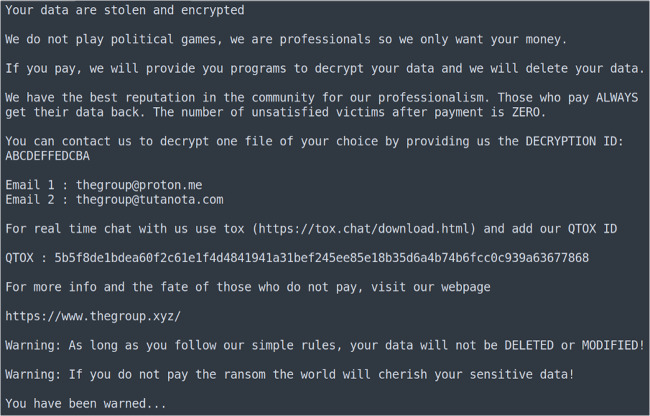
Ransomware attack notification. This message was displayed on a Notepad window on screens of Ward@Home computer terminals.

Ward@Home operators are noticing malfunctions in their systems. The swift intervention of a technician found that some of the servers and some hosts in Ward@Home that were open had a Notepad window on their screens (Fig. 1b) displaying the message of Fig. 2.

### Time: 00:06:51. Ambient temperature: 24 °C

Major incident declared to TradiCare and CommiNet by Operational Director (OD) ‘Ward@Home’. Major Incidents not transmitted to staff or patients as servers are locked. The OD telephones the executive leadership teams and all staff for whom he has personal contact details, asking them to call all their own staff contacts and to share patient contact details. An Incident Room (IR) is established according to the Ward@Home cybersecurity procedures (Fig. 1c).

**Fig. 3:**
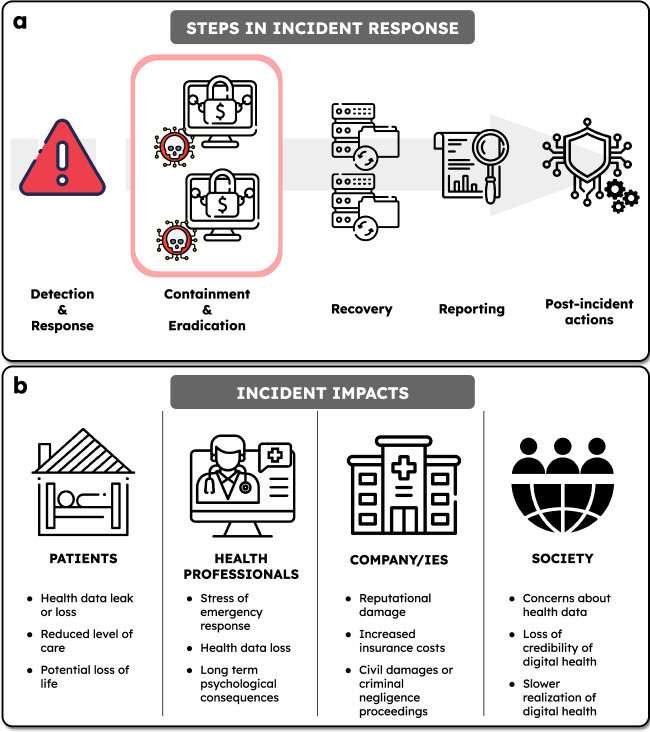
Summary of the response to and impacts of a cybersecurity incident of the type described. **a** The steps to be undertaken in response to a cybersecurity incident. **b** Impacts of the cybersecurity attack on patients, health professionals, companies, and society.

The Ward@Home team tries to revert to backups; however, they realize that the short-term backup servers have been compromised. The offline backup could be used to recover most of the users’ data and is 2 weeks old. The time needed to re-install the servers and restore the backups is estimated at 2 days. The ‘Ward@Home’ services will not be available during this time (Fig. 3).

### Time: 00:07:49. Ambient temperature: 27 °C

Summary of OD status report to IR:

Present: all contactable OC staff, CEO TradiCare, VP RQC CommiNet“

1. *No communication possible via ‘Ward@Home’ platform to staff or patients*
2. *All ‘Ward@Home’ patients and Operations Room phones are locked*.
3. *Available Incident Room staff: 37*.
4. *Available Community care staff: 93, with efforts made to contact more*.
5. *Staff communications are only possible via mobile phones and WhatsApp*.
6. *Lists of patients under critical care and patient addresses are encrypted: IR whiteboard created*.
7. *All contactable staff have been asked to share known vulnerable patients and their addresses and mobile numbers, if known, by WhatsApp.”*

Question: OD to Clinical Duty Lead: ‘Ward@Home’: *“How many Ward@Home patients need urgent intervention or hospitalization per day.” Answer: Normally, 3%, which rose to 9% in the heat wave of July 2023, but then we had no patients in the ‘*close observation’ status. *With this afternoon’s predicted temperature of 46* °C*, the best guess is 800 patients, whom we cannot identify, contact, or effectively triage according to risk. Initial reports indicate that breathing and pulse oximetry monitors are not communicating with the patient consoles, and neither patients nor staff are being alerted to worsening clinical conditions. Patients are finding it extremely difficult to contact us because the telephone lines are jammed and there are long delays in getting ambulances to them due to the heat wave and response to unrelated emergency calls. If the situation is not resolved before peak afternoon temperatures are reached, we anticipate many deaths in our patient group directly attributable to the attack. There is also a substantial data breach, and we will make a full assessment of this within a 48-hour window.”*

## Summary

We address the question: when the HaH meets a systemic ransomware attack (or other similar cybersecurity or major network event cueing massive outage), can that be labeled a Black Swan event^10^, i.e., an unpredictable event that is beyond what is typically expected of a situation and has potentially severe consequences? This article predicts such events, so by definition, they cannot be labeled as unpredictable ‘Black Swans’ in the future. This article is not designed to create alarm or to hinder the development of the HaH model, but rather to highlight the need for careful risk assessment, planning, monitoring, and defense-in-depth approaches to protect this care model against cybersecurity vulnerabilities^11^. If a HaH system is vulnerable to attack, this would turn the very scalability and inherent benefit of the system on its head resulting in a systematic incident involving many patients. Large cybersecurity incidents that cripple critical infrastructure, including health system infrastructure, are no longer rare^12,13^. Identified healthcare system cybersecurity vulnerabilities, which are also components of HaH systems include cyber-medical systems, electronic consultation services, and Internet of Medical Things (IoMT) devices, particularly when these systems are not updated with security patches The former two systems are vulnerable to ransomware and denial of service (DoS) attacks, while IoMT is vulnerable to DoS attacks^12,13^. Recent policy announcements and guidelines recognize the criticality of the cybersecurity challenge in medicine^14^. In the healthcare sector between 2018 and 2022, there has been a 278% increase in large cybersecurity breaches involving ransomware^15^. Actions that can be taken by to protect HaH systems include the careful identification of attack modes, the redesign of the entire system networked systems adopting defense in depth and zero trust principles (a series of layered defensive mechanisms designed to protect health data and systems with deeper layers protecting if higher layers succumb to attack)^16^, choice of IoMT subcomponents developed according to the best practices of design for resilience to cybersecurity threats^14^ IoMT ‘fleet’ oversight management with risk assessed approaches for over-the-air patching of IoMT device vulnerabilities^16^, and adequate and repeated training for personnel and, if needed, patients^12,13^. If a HaH system succumbs to a situation as severe as the fictional one described above, the consequences we described are realistic, patient deaths and severe harm, the inability of clinicians to deliver care, and a loss of confidence and trust in healthcare systems; setting aside criminal proceedings for failing to protect patients under their care.

Cybersecurity risks in the healthcare system that are now developing around digital technologies, particularly as they relate to HaH approaches, should be recognized as critical infrastructure risks that could in themselves cause a major public health emergency. These risks no longer just relate to data loss or danger to the individual patient and should be placed on the highest level of priority with the introduction of new modes of networked care delivery, and budgets should be prioritized for their delivery.

## References

[1] Deloitte. *Hospital at Home*. *A Model with a Future* (Deloitte, 2022).

[2] British Geriatrics Society. *Bringing Hospital Care Home: Virtual Wards and Hospital at Home for Older People (Position Statement)* (British Geriatrics Society, 2022).

[3] Lasserson D, Cooksley T (2023). Virtual wards: urgent care policy must follow the evidence. BMJ.

[4] Gilbert, S. Digital respiratory healthcare regulation (eds Pinnock, H., Poberezhets, V. & Drummond, D.). In *Digital Respiratory Healthcare* (European Respiratory Society, 2023).

[5] Hakim, R. Realising the potential of virtual wards. (2023).

[6] Greenhalgh T (2021). Remote management of covid-19 using home pulse oximetry and virtual ward support. BMJ.

[7] O’Malley E-J, Hansjee S, Abdel-Hadi B, Kendrick E, Lok S (2022). A Covid-19 virtual ward model: a preliminary retrospective clinical evaluation from a UK district general hospital. J. Prim. Care Community Health.

[8] McGlave CC, Nikpay SS, Henning-Smith C, Rydberg K, Neprash HT (2023). Characteristics of short-term acute care hospitals that experienced a ransomware attack from 2016 to 2021. Health Aff. Sch.

[9] McGlave, C. C., Neprash, H. & Nikpay, S. *Hacked to Pieces*? *The Effects of Ransomware Attacks on Hospitals and Patients*. SSRN Scholarly Paper 10.2139/ssrn.4579292 (2023).

[10] Taleb, N. N. *The Black Swan* (Random House, 2009).

[11] Medicine TLR (2021). Digital health: balancing innovation and cybersecurity. Lancet Respir. Med..

[12] Alawida M, Omolara AE, Abiodun OI, Al-Rajab M (2022). A deeper look into cybersecurity issues in the wake of Covid-19: a survey. J. King Saud Univ.—Comput. Inf. Sci..

[13] He Y, Aliyu A, Evans M, Luo C (2021). Health care cybersecurity challenges and solutions under the climate of COVID-19: scoping review. J. Med. Internet Res..

[14] US FDA. *Cybersecurity in Medical Devices: Quality System Considerations and Content of Premarket Submissions Guidance for Industry and Food and Drug Administration Staff* (US FDA).

[15] Healthcare Sector Cybersecurity. I*ntroduction to the Strategy of the U.S. Department of Health and Human Services* (Healthcare Sector Cybersecurity, 2023).

[16] Sun Y, Lo FP-W, Lo B (2019). Security and privacy for the internet of medical things enabled healthcare systems: a survey. IEEE Access.

